# Time to SARS‐CoV‐2 clearance in African, Caucasian, and Asian ethnic groups

**DOI:** 10.1111/irv.13238

**Published:** 2024-06-04

**Authors:** Louis Sides Ndjengue Nson, Daniella Ndombi Delpo Dede, Fabrice Lotola Mougeni, Natacha Bouassa, Basma Bennjakhoukh, Alexandra Luthi, Anselme Voubou, Juliette Atatama, Raymond Tat Pambou, Guy Dieudonné Mvogo, Victorien Sah, Bertin Atangana, Amandine Mveang Nzoghe, Anicet Christel Maloupazoa Siawaya, Paulin N. Essone, Pélagie Mougola Bissiengou, Bénédicte Ndeboko, Joel Fleury Djoba Siawaya

**Affiliations:** ^1^ Service Laboratoire Clinique Aries Medicus Port‐Gentil Gabon; ^2^ Department of Public Health, Faculty of Health Sciences Kesmonds International University Bamenda Cameroon; ^3^ Centre de Recherches Médicales de Lambaréné (CERMEL) Lambarene Gabon; ^4^ Faculty of Science University of the Witwatersrand Johannesburg South Africa; ^5^ CHU Mère‐Enfant Fondation Jeanne EBORI Libreville Gabon; ^6^ Service d'Immunologie, Département des Sciences Fondamentales, Faculté de Médecine Université des Sciences de la Santé Libreville Gabon; ^7^ Département de Biologie Cellulaire et Biologie Moléculaire Université des Sciences de la Santé Libreville Gabon; ^8^ Unité de Recherche et de Diagnostics Spécialisés Laboratoire National de Santé Publique Libreville Gabon

**Keywords:** COVID‐19, ethnicity, herd immunity, RT‐PCR, SARS‐CoV‐2 clearance, vaccination

## Abstract

**Background:**

COVID‐19 may become a seasonal disease. SARS‐CoV‐2 active circulation coupled with vaccination efforts has undoubtedly modified the virus dynamic. It is therefore important investigate SARS‐CoV‐2 dynamic in different groups of population following the course of spatiotemporal variance and immunization.

**Methods:**

To investigate SARS‐CoV‐2 clearance in different ethnic groups and the impact of immunization, we recruited 777 SARS‐CoV‐2‐positive patients (570 Africans, 156 Caucasians, and 51 Asians). Participants were followed and regularly tested for 2 months until they had two negative tests.

**Results:**

The vaccination rate was 64.6%. African individuals were less symptomatic (2%), Caucasians (41%) and Asians (36.6%). On average, viral clearance occurred after 10.5 days. Viral load at diagnosis was inversely correlated with viral clearance (*p* < 0.0001). The time of SARS‐CoV‐2 clearance was higher in Africans and Caucasians than in Asians (Dunn's test *p* < 0.0001 and *p* < 0.05, respectively). On average, viral clearance occurred within 9.5 days during the second semester (higher rate of vaccination and SARS‐CoV‐2 exposition), whereas it took 13.6 days during the first semester (lower rate of vaccination and SARS‐CoV‐2 exposition) (Mann–Whitney *t*‐test *p* < 0.0001).

**Conclusion:**

In conclusion, ethnicity and spatiotemporal changes including SARS‐CoV‐2 exposition and immunization affect SARS‐CoV‐2 clearance.

## 1 INTRODUCTION

In 2019, a new coronavirus (SARS‐CoV‐2) firstly reported in Huwan in China, causing an epidemic of severe pneumonia poorly known to most practitioners and researchers.^1^ The disease rapidly spread from China to the rest of the world, becoming a pandemic.^2^, ^3^ To date, the world experienced four waves driven by SARS‐CoV‐2 variants. However, Africa has gone through all these waves less impacted than other regions of the world.^4^, ^5^, ^6^

Data show that all continents and countries are unequal regarding COVID‐19 epidemic profiles.^5^ Also, it has been suggested that ethnicity might also influence disease course.^7^, ^8^ Understanding why the virus spreads at different speeds and affects populations differently is essential. The main argument for Africa's resilience has been the younger age of its population. Also, it has been hypothesized that the high exposure of Africans to the diversity of pathogens in Africa increases the population's probability of being exposed to cross‐protective epitopes. A recent study showed pre‐existing cross‐reactive humoral immunity to SARS‐CoV‐2 in the Gabonese population,^9^ Tanzanian, and Zambian.^10^

SARS‐CoV‐2 immunity produced following infection or vaccination is critical for controlling the spread of COVID‐19. In Gabon between April to September 2021 (Semester 1 [S1]), up to 36% of the population showed signs of immunization,^11^, ^12^ and between October 2021 to March 2022 (Semester 2 [S2]), 87% of the populations tested positive for anti‐SARS‐CoV‐2 antibodies.^6^ The first the part of S1 was characterized by the circulation of the alpha (α) variant (B.1.1.7 [40%]), the eta (η) variant (B.1.525 [32%]) and to a lesser extend beta (β) variant (B.1.351 [6%]).^13^ By the end of S1, the beta (β) variant represented over 50% of circulating variants (country data). During S2, the circulation of the omicron (Ο) variant drove the pandemic and was virtually the only circulating variant from the second half of S2 (country data estimates [not published]). Here, we investigate SARS‐CoV‐2 spatiotemporal variance, immunization and viral clearance in Black African, White European‐Americans, and Asians in the Gabonese context.

## 2 METHODS

The study took place between April 2021 and March 2022 in Port‐Gentil, Gabon. The study period was divided into two semesters. S1 covered April to September 2021, and S2 went from October 2021 to March 2022. The Gabon population immunization rate (natural and/or vaccinated) was estimated between 13% and 36% for the S1^11^, ^12^ and 87% for the S2.^6^

Participants were recruited in the setting of Aries Medicus Clinic and its associated containment sites. Participants included in the study were participants tested regularly as required by their employer's policy (workers from oil companies and other office workers). They were recruited from an active surveillance process rather than a passive one, minimizing the bias due to the time delay from infection to diagnostic testing. Study participants were symptomatic or non‐symptomatic patients with a positive test with PCR cycle threshold values (Ct) < 30 and who were PCR‐tested regularly (Days 2, 3, 5, 7, 8, 10, 12, 14, 16, 20, 22, 30, 40, and 50) until they had two negative tests.

COVID‐19‐positive patients followed an anti‐COVID‐19 treatment protocol, which consisted of the combined combination of azithromycin (500 mg first day and 250 mg from the second day for 5 days), amoxicillin plus clavulanic acid 2 g/day (for 7 to 10 days), vitamin C (1000 mg per day for 10 days), zinc tablet (15 mg per day for 10 days), and vitamin D (100,000 IU in a single dose). The treatment was adjusted based on body mass index (BMI) when required.

### 2.1 Collection of data

For each participant, we collected sociodemographic data (gender, age, occupation, type of screening, etc.) and clinical data (fever, cough, fatigue, headaches, sore throats, diarrhea, anosmia, ageusia, etc.). Also, we recorded information on samples collected (nasopharyngeal, oropharyngeal, etc.) and vaccination status. Patients were defined as vaccinated if they were fully vaccinated (received all the vaccine doses recommended). At each visit, participants were re‐tested, and the results were recorded. We also recorded patients' COVID‐19 vaccination status (type of vaccine included).

### 2.2 PCR testing

The samples of nasopharyngeal and oropharyngeal swabs were collected from each participant and treated under a Class 2 biology safety cabinet. ARN extraction was done using Bioer's automatic extractor (Bioer Technology Co. Ltd, China) and the Bioflux kits (Fluxion Biosciences, USA). The amplification was done using the Bioflux kits on the Bioer LineGene 9600 Plus thermal cycler. All experiments were done strictly according to the manufacturer's instructions. The results expressed in the Ct value of the N and ORFab genes were recorded.

### 2.3 Time to viral clearance

We define as time to viral clearance the lapse of time between the first SARS‐CoV‐2 positive PCR and the two consecutive and independent SARS‐CoV‐2 negative PCR test.

### 2.4 Statistics

The statistical analysis was performed using GraphPad Prism software version 6. Linear regression and correlation analysis were done to investigate the link between viral quantum and time to negativity. In addition, we used the ANOVA one‐way non‐parametric multiple comparisons tests (Kruskal–Wallis test) coupled with Dunn's multiple comparisons test to compare the groups. Multivariate analysis was used to control confounding effects. For longitudinal comparisons we used the two‐way ANOVA, multiples comparisons test. Contingency tables were used to determine the association between variables. The threshold of significance was a *p* value below 0.05. Analysis of interactions between the categorical independent variables was done using multivariate analysis.

### 2.5 Ethics considerations

Informed consent was obtained from all participants. The CHU Mère‐Enfant Fondation Jeanne EBORI Scientific board approved this study.

## 3 RESULTS

A total of 777 patients were included in the study (570 Black Africans, 156 Caucasians [Whites from Europe and the United States], and 51 Asians). Seven hundred and twenty‐nine (94%) patients had all required data available (ethnics, vaccines status, clinical presentation, all SARS‐CoV‐2 PCR results, and time to viral clearance) (Table 1). The average age was 41.16 years (median age 41 years). The F/M sex ratio was 0.3. The median time to viral clearance in the population was 10 days (the average time to negativity was 10.5 days). The age distribution was similar in all ethnic groups (Tables 1 and 2).

**TABLE 1:** Distribution and characteristics of the studied population‐based ethnics, vaccine status, and COVID‐19 clinical presentation.

Characteristic	*N* = 777^a^
Symptoms	
No	546 (70%)
Yes	231 (30%)
Semesters	
1	152 (20%)
2	625 (80%)
Age (median—all)	41 (CI: 40–42)
Age (median—Africans)	41 (CI: 40–41)
Age (median—Asians)	42 (CI: 39–47)
Age (median—Caucasians)	43 (CI: 40–45)
Gender	
Female	178 (23%)
Male	599 (77%)
Time to viral clearance (median)	10.0 (7.0, 14.0)
Unknown	9
Ethnics	
Africans	570 (73%)
Asians	51 (6.6%)
Caucasians (Americans)	14 (1.8%)
Caucasians (Europeans)	142 (18%)
Vaccinated	
No	275 (35%)
Yes	502 (65%)

^a^ *n* (%); median (IQR).

**TABLE 2:** Populations' ethnicity‐based sizes and age distribution and vaccination rate.

	All	Africans (Black)	Caucasians (White)	Asians
Gender	Males: 77%	Males: 78.25%	Males: 72.4%	Males: 87.7%
	Females: 23%	Females: 21.75%	Females: 27.6%	Females: 12.3%
Median age [25% to 75% percentile]	41 [35–48]	41 [35–46]	43 [31.25–53]	42 [33–53]
Mean age (95% CI)	41.16 (40.3–42)	41.03 (40.2–42)	40.9 (38.5–43.4)	43.3 (39.7–46.9)
Vaccination rate	65%	52%	100%	100%

### 3.1 The vaccination rate in the studied population

Overall, 65% of patients in the study were vaccinated. About 52% of Black Africans were vaccinated (Table 2). All Caucasians and Asians were vaccinated (vaccination rate 100%). Johnson, Sinopharm, and Pfizer vaccines were the most represented in the population of vaccinated patients, with 38.4%, 24.2%, and 23.5%, respectively; 37.5% of individuals recruited in S1 were vaccinated, whereas 77.3% of recruited in S2 were vaccinated.

### 3.2 Clinical manifestation in vaccinated versus non‐vaccinated individuals

Analyzing all participants (from all ethnicity), we found no significant differences between vaccinated and non‐vaccinated individuals. In the African population, only 2% of vaccinated individuals were symptomatic (98% of asymptomatic). In vaccinated Caucasians and Asians, respectively, 41% and 36.6% of individuals were symptomatic.

Vaccinated Africans were more likely to be asymptomatic when compared with Caucasian (chi‐square: 92.6; odds: 37.15; *p* value <0.0001) and Asians (chi‐square: 61; odds: 30.7; *p* value <0.0001). We observed no significant differences between Asians and Caucasian.

### 3.3 ORFab Ct values of participants

**FIGURE 1:**
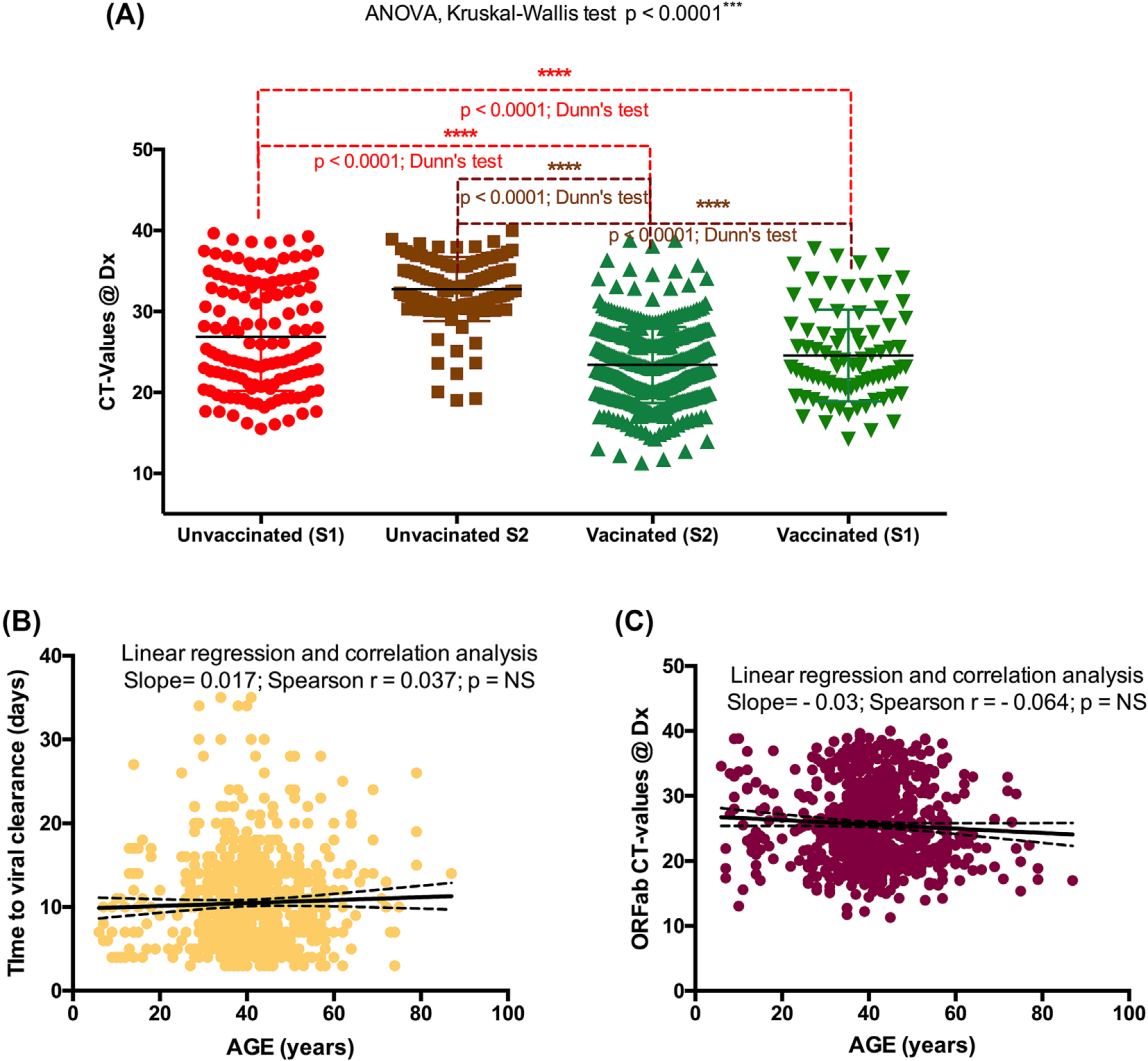
(A) Analysis of variance: Ct values at diagnosis significantly differed between the ethnical groups (ANOVA, Kruskal–Wallis test *p* < 0.0001). It was lower in unvaccinated in Semester 1 (S1) versus unvaccinated in Semester 2 (S2) (Dunn's test *p* < 0.0001). Ct values at diagnosis of unvaccinated individuals during S2 were significantly higher than vaccinated individuals during S1 and S2 (Dunn's test *p* < 0.0001). Also, Ct values of unvaccinated individuals during S1 were significantly higher than vaccinated individuals during S1 and S2 (Dunn's test *p* < 0.0001). (B,C) Linear regression and correlation: (B) time to viral clearance based on age and (C) ORFab Ct values based on age. The observation was not significant (NS).

The analysis of participants ORFab Ct values at diagnosis showed that unvaccinated peoples had significantly higher ORFab Ct values than vaccinated peoples (both in S1 and S2) (*p* value <0.0001) (Figure 1A). When comparing participants from S1 and S2, irrespectively of vaccination status, we found no significant differences.

### 3.4 Age based correlation (with viral clearance and ORFab Ct values)

Linear regression and correlation analysis showed no correlation between time to viral clearance and age (Figure 1B). Also, no correlation was found between ORFab Ct values at diagnosis and age (Figure 1C).

### 3.5 Correlation between time to viral clearance and ORFab Ct values

**FIGURE 2:**
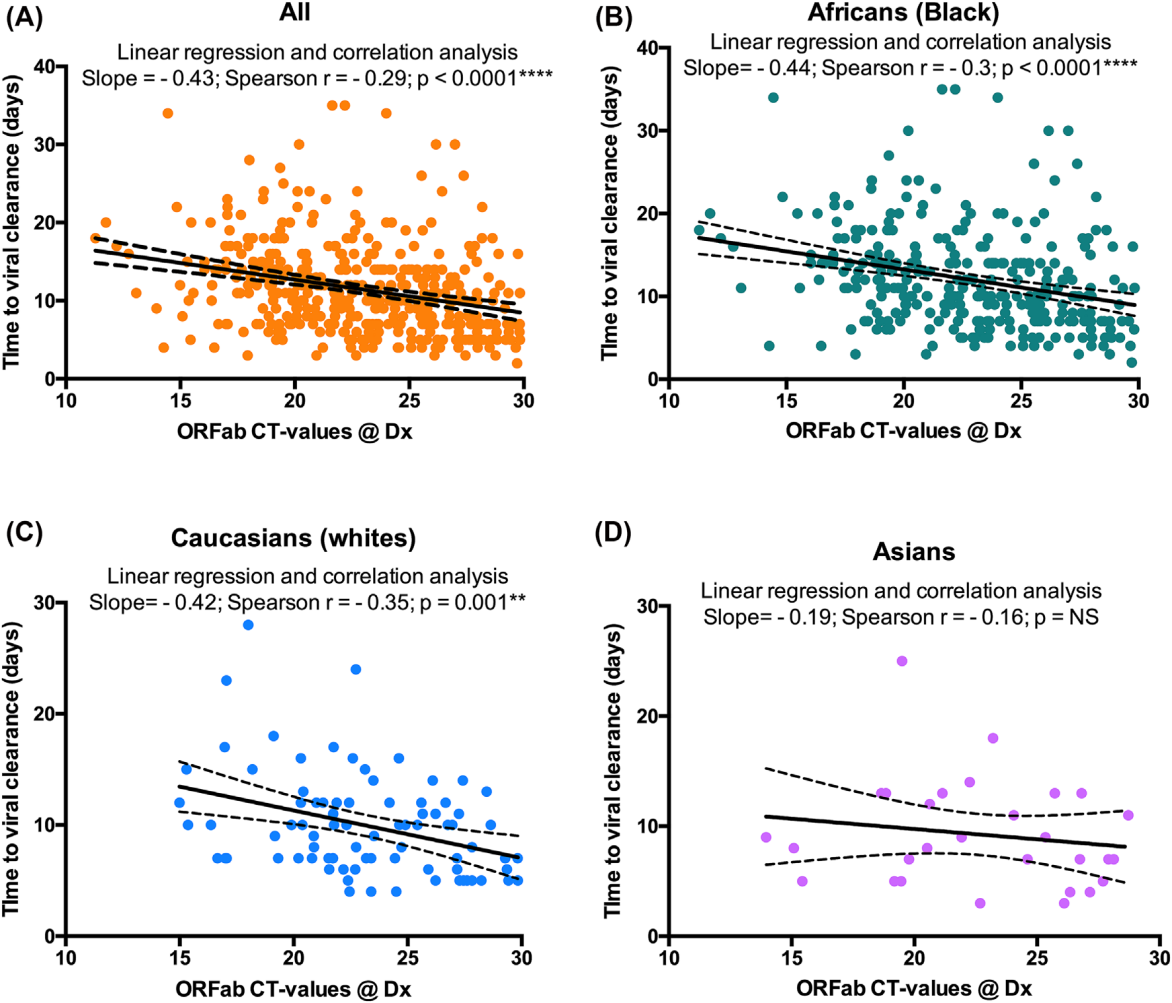
Linear regression and correlation between time to viral clearance and ORFab Ct values. (A) All participants: a significant inversed correlation between ORFab Ct values at diagnosis and time to viral clearance (slope = −0.43, *r* = −0.29, *p* < 0.0001) was observed. (B) Black African population: the inversed correlation between ORFab Ct values at diagnosis and time to viral clearance was significant (slope = −0.44, *r* = −0.3, *p* < 0.0001). (C) Caucasian population: inversed correlation between ORFab Ct values at diagnosis and time to viral clearance is significant (slope = −0.42, *r* = −0.35, *p* < 0.0001) was significant. (D) Asian population: no significant correlation was observed (slope = −0.19, *r* = −0.16, *p*: not significant [NS]).

Linear regression and correlation analysis showed a significant inversed correlation between ORFab Ct values at diagnosis and time to viral clearance (slope = −0.43, *r* = −0.29, *p* < 0.0001) (Figure 2A). Analyzing ethnical groups separately the correlation between ORFab Ct values at diagnosis and time to viral clearance remained significant for Africans (slope = −0.44, *r* = −0.3, *p* < 0.0001) and Caucasians (slope = −0.42, *r* = −0.35, *p* < 0.0001) only (Figure 2B–D).

### 3.6 ORFab Ct values and time to viral clearance in Africans, Caucasians, and Asians

**FIGURE 3:**
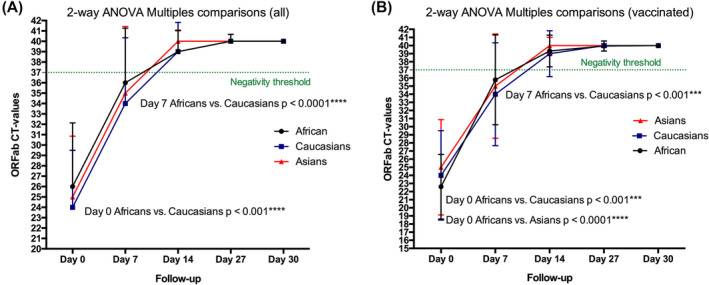
Longitudinal measures of ORFab Ct values in Africans, Caucasians, and Asians: (A) all participants (vaccinated and unvaccinated). At diagnosis and a week after, ORFab Ct values were significantly lower in Africans compared with Caucasians (two‐way ANOVA, multiples comparisons test; diagnosis *p* < 0.001; Day 7 *p* < 0.0001). (B) Only vaccinated participants. At diagnosis and at Day 7, ORFab Ct values were significantly lower in in Africans compared with Caucasians and Asians (Africans vs. Caucasians *p* < 0.001; Africans vs. Asians *p* < 0.0001).

**FIGURE 4:**
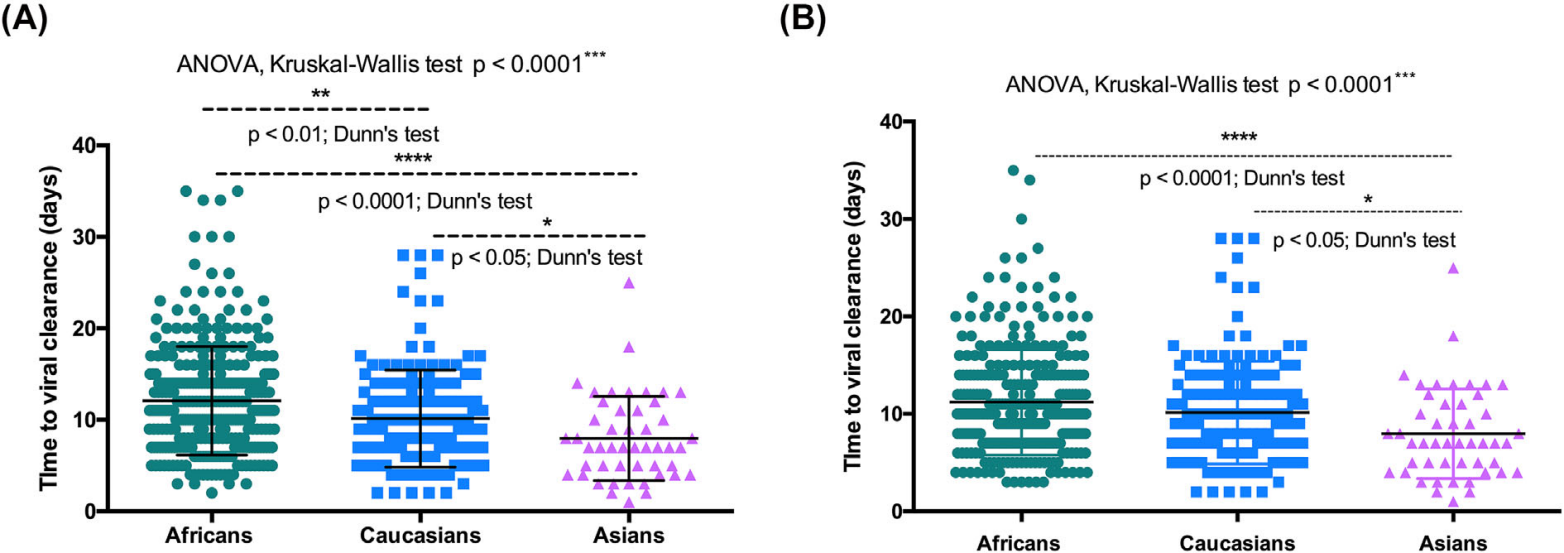
(A) The time to viral clearance significantly differed between the ethnical groups (ANOVA, Kruskal–Wallis test *p* < 0.0001). It was higher in Africans versus Caucasians (Dunn's test *p* < 0.01) and Asians (Dunn's test *p* < 0.0001). The time to viral clearance was also higher in Caucasians versus Asians (Dunn's test *p* < 0.05). (B) After correction for unvaccinated: no differences in time to viral clearance between Black Africans and Caucasians. The time to viral clearance of Africans and Caucasians remained significantly higher than that of Asians (Dunn's test *p* < 0.0001 and *p* < 0.05, respectively).

Comparing ORFab Ct of Africans, Caucasians, and Asians, our analysis showed that at diagnosis and a week after, the viral load (as indicated by the lower Ct value) was higher in Africans compared with Caucasians and Asians (two‐way ANOVA, multiple‐comparison test; Africans vs. Caucasians *p* < 0.001; Africans vs. Asians *p* < 0.0001) (Figure 3A,B). The time to viral clearance was significantly different between the ethnical groups (ANOVA, Kruskal–Wallis test *p* < 0.0001). It was significantly higher for African as compared with Caucasians (Dunn's test *p* < 0.01) and Asians (Dunn's test *p* < 0.0001) (Figure 4A). Also, the time to viral clearance was significantly higher for Caucasians than Asians (Dunn's test *p* < 0.05) (Figure 3B). After correcting for unvaccinated Black Africans and analyzing only vaccinated individuals, the trend changed. No differences in time to viral clearance were observed between Black Africans and Caucasians. However, the time to viral clearance of both Black African and Caucasians remained significantly higher than the one of Asians (Dunn's test *p* < 0.0001 and *p* < 0.05, respectively) (Figure 4B). As for the ANOVA test, after correcting for unvaccinated Black Africans, no differences in time to viral clearance were observed between Black Africans and Caucasians.

Comparing genders, although men seemed to clear the virus more rapidly than women (non‐vaccinated: 9.7 days vs. 12 days; vaccinated: 10.6 days vs. 11 days), the differences did not reach statistically significant.

### 3.7 Viral clearance, clinical manifestation, and vaccine status

Mann–Whitney test and the unpaired *t*‐test‐based analysis showed there is no significant difference in the time to viral clearance between symptomatic (median: 10 [CI: 10–11]; mean 11.1 [CI: 10.37–11.78]) and asymptomatic (median: 10 [CI: 9–10]; mean 10.6 [CI: 10.13–11.09]) participants when all participants are considered. Looking only at vaccinated individuals' data analysis showed no significant difference in the time to viral clearance between symptomatic (median: 10 [CI: 9–11]; mean 10.87 [CI: 9.71–12.04]) and asymptomatic (median: 10 [CI: 9–10]; mean 10.75 [CI: 10.18–11.32]) participants.

### 3.8 Effect of immunity on viral clearance

**FIGURE 5:**
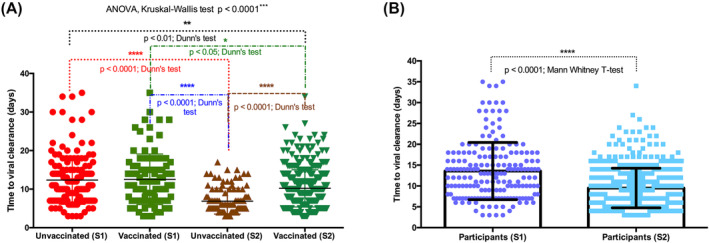
Effect of vaccines and group immunity on time to viral clearance. (A) Semester 1 (S1: lower vaccination rate and low SARS‐CoV‐2 exposition): no significant difference between vaccinated and non‐vaccinated individuals. (B) Semester 2 (S2: higher vaccination rate and high SARS‐CoV‐2 exposition [87%]): time to viral clearance decreased significantly (ANOVA, Kruskal–Wallis test *p* < 0.0001). The time to viral clearance of S2‐non‐vaccinated individuals was significantly lower than that of S1‐non‐vaccinated individuals (Dunn's post‐test *p* < 0.0001). Also, the time to viral clearance of S2‐vaccinated individuals was significantly lower than that of S1‐vaccinated individuals (Dunn's post‐test *p* < 0.05).

In S1 of our study (S1: lower number of vaccinated individuals and an estimated low SARS‐CoV‐2 exposition), no significant difference in the time to viral clearance was observed between vaccinated and non‐vaccinated individuals. In the second semester of our study (higher number of vaccinated individuals and an estimated high SARS‐CoV‐2 exposition [87%]), the time to viral clearance decreased significantly (ANOVA, Kruskal–Wallis test *p* < 0.0001) (Figure 5A). The time to viral clearance of S2‐non‐vaccinated individuals was significantly lower than the one of S1‐non‐vaccinated individuals (Dunn's post‐test *p* < 0.0001). Also, the time to viral clearance of S2‐vaccinated individuals was significantly lower than that of S1‐vaccinated individuals (Dunn's post‐test *p* < 0.05). During S2, the times of viral clearance of non‐vaccinated individuals were significantly lower than the ones of vaccinated individuals (Dunn's post‐test *p* < 0.0001). S1 and S2 aggregated analysis (irrespective of vaccination status) showed that viral clearance was significantly faster during the second semester (9.5 days on average) than during the first semester (13.6 on average) (Figure 5B) (Mann–Whitney *t*‐test *p* < 0.0001).

### 3.9 Multivariate analysis

Looking at interactions between the categorical variables with the time to viral clearance set as the outcome. The analysis of interactions between the categorical independent variables (ethnicity, clinical representation, and vaccine status) showed no significant interaction between them. Adding semesters to this model increased the fit of the model, making it the best model, as indicated by the Akaike information criterion (AIC) (Tables 3 and 4).

**TABLE 3:** Multivariate regression analysis of factors associated with time to viral clearance.

Characteristic	Univariable analysis			Model without semester—log transformation			Model with semester—log transformation			
	*N*	Estimated change (%)	95% CI	*p* value	Estimated change (%)	95% CI	*p* value	Estimated change (%)	95% CI	*p* value
Vaccinated	768									
No		—	—		—	—		—	—	
Yes		9	1–17	0.033	19	8–30	<0.001	35	23–48	<0.001
Symptoms	768									
No		—	—		—	—		—	—	
Yes		8	−1 to 0.17	0.085	9	0.00–19	0.047	8	0–17	0.043
Ethnics	768									
Africans		—	—		—	—		—	—	
American‐Caucasians		20	−10 to 60	0.23	11	−17 to 48	0.51	1	−23 to 34	0.93
Asians		−29	−39 to 17	<0.001	−34	−45 to 23	<0.001	−38	−47 to 28	<0.001
European‐Caucasians		−7	−16 to 3	0.20	−15	−24 to 5	0.005	−25	−33 to 16	<0.001
Semesters	768									
1		—	—					—	—	
2		−32	−39 to −26	<0.001				−39	−4 to 33	<0.001
AIC					1249			1154		
Deviance					225			198		

**TABLE 4:** Multivariate regression analysis of factors associated with time to viral clearance (including categorical variable interaction).

Characteristic	Clinical symptoms versus semesters			Interaction symptoms versus ethnics			Interaction vaccine status versus semesters			Interaction vaccine status versus semesters versus clinical symptoms		
	Change in %	95% CI	*p* value	Change in %	95% CI	*p* value	Change in %	95% CI	*p* value	Change in %	95% CI	*p* value
Vaccinated												
No	—	—		—	—		—	—		—	—	
Yes	35	23–48	<0.001	35	25–48	<0.001	6	−11 to 27	0.53	−2	−21 to 22	0.87
Ethnics												
Africans	—	—		—	—		—	—		—	—	
Americans	1	−23 to 34	0.93	11	−21 to 52	0.56	8	0–17	0.062	4	−21 to 36	0.78
Asians	−38	−47 to 28	<0.001	−44	−53 to 32	<0.001				−37	−46 to 27	<0.001
Europeans	−25	−33 to 16	<0.001	−30	−39 to 20	<0.001	—	—		−22	−30 to 13	<0.001
Semesters							4	−21 to 36	0.78			
1	—	—		—	—		−37	−46 to 27	<0.001	—	—	
2	−39	−46 to 32	<0.001	−39	−45 to 34	<0.001	−22	−30 to 13	<0.001	−49	−56 to 41	<0.001
Symptoms												
No	—	—		—	—		—	—		—	—	
Yes	7	−10 to 28	0.43	3	−7 to 14	0.55	−47	−53 to 39	<0.001	−3	−23 to 23	0.81
AIC	1156			1152			1147			1151		
Variable interactions			Not significant			Not significant			Significant			Not significant

The multivariate analysis in its semester included model revealed an association between clinical presentation and viral clearance. The time to viral clearance increased by 8% when symptomatic (*p* value = 0.043) (Table 3). Also, multivariate analysis showed that the viral clearance of vaccinated individuals increased by 35% compared with unvaccinated (*p* value <0.001) (Table 3). The multivariate analysis confirmed that the time to viral clearance was significantly different between the ethnical groups (significantly higher for African as compared with European‐Caucasians [*p* < 0.001] and Asians [p < 0.001]; Tables 3 and 4). The analysis also confirmed that viral clearance was significantly faster during S2 than during S1 (*p* < 0.001) (Tables 3 and 4).

## 4 DISCUSSION

The present study is one of the first evaluated in the same setting, times to SARS‐CoV‐2 negativity in populations of Africans, Caucasians, and Asians, taking into account natural exposition and vaccination to SARS‐CoV‐2.

Our study showed a significant inversed correlation between ORFab Ct values at diagnosis and time to negativity, which is expected; clearing of high viral loads should take more time than clearing low viral loads.^14^ The raw analysis of ORFab Ct and viral clearance between ethnicities showed that Asians and Caucasians who had significantly lower viral load at diagnosis cleared the virus significantly faster than Africans. As all Asians and Caucasians were vaccinated, we corrected for unvaccinated Africans. After correction, Asians (with an average of 8 days) still cleared the virus significantly faster than Caucasians (10 days) and Africans (11 days). It appears that initial viral load and ethnicity would influence virus clearance. Surprisingly, our data showed that unvaccinated participant cleared the virus faster than vaccinated participant. This probably due to the initial viral load that was low in unvaccinated individuals recruited and for whom we cannot exclude previous SARS‐CoV‐2 natural exposition during S1. However, in our study, the high rate of SARS‐CoV‐2 natural exposition of unvaccinated participant during study S2 (country data)^6^ is less speculative and may explain our observations. Indeed, it has been showed that natural exposition to SARS‐CoV‐2 pre‐variant of concern may influence SARS‐CoV‐2 viral load.^15^ Also, we showed that vaccination and ethnicity influence the occurrence of symptoms. Others have also reported a faster viral clearance time and lower symptoms occurrence in vaccinated individuals than that among unvaccinated.^16^, ^17^ Studies investigating ethnical‐ or racial‐based SARS‐CoV‐2 infection disparities in developed countries principally highlighted the socioeconomic status and health access equity as main drivers of SARS‐CoV‐2 infection ethnical associated disparities.^18^, ^19^, ^20^ Our approach and setting was different and showed that the time to viral clearance is similar for both sub‐Saharan Africans and Caucasians. Also the big parts of SARS‐CoV‐2 infected sub‐Saharan Africans were asymptomatic contrarily to Asians and Caucasians.

Further investigation into the influence of SARS‐CoV‐2 exposition on viral clearance showed that community immunity or herd immunity has a greater impact in reducing time for SARS‐CoV‐2 clearance. Indeed, our study showed a longer time to negativity for vaccinated individuals in S2 as compared with the unvaccinated cases during the same period. This observation may be explained by the higher rate natural immunization during S2. Indeed, the seroprevalence survey (WHO Unity Study) done in Gabon around that period showed an immunization rate of 87%.^6^ Moreover, on average, the time to viral clearance dropped from 13.6 days in S1 to 9.5 days in S2. A previous study done in the Gabonese population during the first year of the pandemic showed a time to viral clearance ranged between 8 and 40 days (17 days on average).^21^ Additionally, as we mentioned in our introductory words, the first part of S1 was characterized by the circulation of the alpha (α) variant, the eta (η) variant, and the beta (β) variant^13^ (country data), whereas in S2, the circulation of the omicron (Ο) variant drove the pandemic with an explosion in the total number of cases that rose by a factor of five (country data estimates). This suggests that, as we went through the different waves of the pandemic, each characterized by increasingly contagious variants that lead to a higher SARS‐CoV‐2 naturally exposed population, the time to SARS‐CoV‐2 clearance decreased, undoubtedly due to the herd effect. Moreover, natural exposition seems to have more effect on time to viral clearance than vaccine. Indeed, the fact that vaccinated individuals in S2 have increased the time to viral clearance than unvaccinated individuals suggests that vaccines were less effective against SARS‐CoV‐2 variants circulating during S2.

## 5 CONCLUSION

Ethnicity and spatiotemporal changes including SARS‐CoV‐2 exposition and immunization affect SARS‐CoV‐2 clearance.

## AUTHOR CONTRIBUTIONS

Louis Sides Ndjengue Nson and Joel Fleury Djoba Siawaya conceived the study, did data analyses, and drafted the first version of the manuscript. Louis Sides Ndjengue Nson, Daniella Ndombi Delpo Dede, Fabrice Lotola Mougeni, Basma Bennjakhoukh, Alexandra Luthi, Anselme Voubou, Juliette Atatama, and Raymond Tat Pambou, Guy Dieudonné Mvogo, Victorien Sah, and Bertin Atangana did data analysis plan and support. Amandine Mveang Nzoghe, Anicet Christel Maloupazoa Siawaya, Pélagie Mougola Bissiengou, Paulin N. Essone, and Bénédicte Ndeboko participated in the study design and support. All authors contributed to the study organization and approved the final manuscript.

## CONFLICT OF INTEREST STATEMENT

The authors declare that they do not have any competing or conflicts of interest.

### PEER REVIEW

The peer review history for this article is available at https://www.webofscience.com/api/gateway/wos/peer-review/10.1111/irv.13238.

## AUTHORS' CONSENT FOR PUBLICATION

All authors read the last version of the manuscript and consented to publication.

## Data Availability

Data can be accessed and made available by contacting the corresponding author by e‐mail (joel.djoba@gmail.com).
